# Supplementary material for: Evaluating the Efficacy of a Peripheral Nerve Simulator-Guided Brachial Plexus Block in Rabbits Undergoing Orthopaedic Surgery Compared to Systemic Analgesia
Source: Vet Sci. 2024 May 13;11(5):213. doi: 10.3390/vetsci11050213 (PMC11125770; doi:10.3390/vetsci11050213)
Supplement: Supplementary file 1 [file vetsci-11-00213-s001.zip › vetsci-2912727-supplementary.pdf]

S1. Post hoc video processing using Python script designed to detect whole rabbit movement and track distance travelled during the first two hours after recovery. Written using Python Software Foundation, version 3.10.5

```python
import csv
import cv2
import logging
import math

video_file_name = "Video_file_name.avi"

# Create a VideoCapture object and read from input file
cap = cv2.VideoCapture(video_file_name)
fps = cap.get(cv2.CAP_PROP_FPS)
frame_count = 0

# Create object detector
object_detector = cv2.createBackgroundSubtractorMOG2(
    history=300,
    varThreshold=30
)

# Setup logging
logging.basicConfig(
    filename=f"{video_file_name}.log",
    encoding="utf-8",
    level=logging.INFO,
    filemode='w'
)
logging.info('Starting script')

# Setup CSV for time/"distance" logging
csv_file = open(
    f'{video_file_name}.csv', 'w',
    encoding='UTF8',
    newline=''
)
csv_writer = csv.writer(csv_file)
csv_writer.writerow(['timestamp', 'cumulative_distance_travelled'])

# Check if video file has opened successfully
if cap.isOpened() is False:
    logging.error('Error opening video file')

previous_detection = None
cumulative_distance_travelled = 0
consecutive_frames_failed_to_read = 0
while cap.isOpened():
    frame_count += 1
```

```python
# Capture frame-by-frame
frame_exists, current_frame = cap.read()

# Get timestamp of frame in seconds
frame_timestamp = frame_count / fps

logging.info(
    f'Frame: {frame_count}. '
    f'Timestamp: {"{:.6f}".format(frame_count / fps)}. '
    f'Exists: {frame_exists}.'
)

if frame_exists is True:
    consecutive_frames_failed_to_read = 0
    height, width, _ = current_frame.shape

    # Extract ROI
    roi = current_frame[0: 720, 0: 576]

    # Object detection
    mask = object_detector.apply(roi)
    _, mask = cv2.threshold(mask, 254, 255, cv2.THRESH_BINARY)
    contours, _ = cv2.findContours(
        mask,
        cv2.RETR_TREE,
        cv2.CHAIN_APPROX_SIMPLE
    )

    # Only storing largest detection (if there is one)
    detection = None
    largest_detected_area = 0
    for cnt in contours:
        # Calculate area and remove small elements
        area = cv2.contourArea(cnt)
        if area > 100 and area > largest_detected_area:
            # cv2.drawContours(roi, [cnt], -1, (0, 255, 0), 2)
            x, y, w, h = cv2.boundingRect(cnt)
            cv2.rectangle(roi, (x, y), (x + w, y + h), (0, 255, 0), 3)

            detection = (x, y, w, h)
            largest_detected_area = area

    if detection:
        logging.info(f'  Detection (x): {detection[0]}.')
        logging.info(f'  Detection (y): {detection[1]}.')
        logging.info(f'  Detection (w): {detection[2]}.')
        logging.info(f'  Detection (h): {detection[3]}.')
```

```
            # Calculate distance moved from last detection
            if previous_detection:
                (
                    previous_x,
                    previous_y,
                    previous_w,
                    previous_h
                ) = previous_detection
                previous_center_point = (
                    previous_x + previous_w / 2,
                    previous_y + previous_h / 2
                )

                (current_x, current_y, current_w, current_h) = detection
                current_center_point = (
                    current_x + current_w / 2,
                    current_y + current_h / 2
                )

                x_difference = abs(
                    current_center_point[0] - previous_center_point[0]
                )
                y_difference = abs(
                    current_center_point[1] - previous_center_point[1]
                )
                distance_travelled = math.sqrt(
                    x_difference ** 2 + y_difference ** 2
                )
                cumulative_distance_travelled += distance_travelled

            previous_detection = detection

        # Display ROI, frame and mask
        cv2.imshow("ROI", roi)
        cv2.imshow("Frame", current_frame)
        cv2.imshow("Mask", mask)
        cvs.waitKey(10)
    else:
        # Failed to read frame
        consecutive_frames_failed_to_read += 1
        if consecutive_frames_failed_to_read >= 30:
            logging.error(
                'Encountered 30 frames in a row that were unreadable, exiting'
            )
            break

    # Always write frame to CSV
```

```
    csv_writer.writerow([
        "{:.6f}".format(frame_count / fps),
        "{:.6f}".format(cumulative_distance_travelled)
    ])

# When everything done, release the video capture object
cap.release()

# Closes all the frames
cv2.destroyAllWindows()

# Close CSV file
csv_file.close()

logging.info('End of script')
```
